# Hepatic Resection is Safe for Metachronous Hepatic Metastases from Ovarian Cancer

**DOI:** 10.7497/j.issn.2095-3941.2012.03.005

**Published:** 2012-09

**Authors:** Guang-cai Niu, Chang-ming Shen, Wei Cui, Qiang Li

**Affiliations:** Department of Hepatobiliary Surgery, Tianjin Medical University Cancer Institute and Hospital, Tianjin 300060, China

**Keywords:** ovarian cancer, liver metastasis, hepatectomy

## Abstract

**Objective:**

To explore the efficacy of hepatic resection (HR) in a relatively unselected group of patients with ovarian cancer liver metastases (OCLM).

**Methods:**

A study was conducted between September 2000 and September 2011 on 60 ovarian cancer patients with hepatic metastases (24 solitary and 36 multiple), 40 of whom had extrahepatic metastases. HR was done in all patients provided that curative hepatic resection was feasible, and extrahepatic disease was controlled with medical and/or surgical therapy.

**Results:**

Most patients (*n*=54; 90.0%) had a negative hepatic margin (R0), whereas 6 patients (10.0%) had microscopic disease at the margin (R1). The prognostic value of each study variable was assessed using log rank tests for univariate analysis and Cox proportional hazard models for multivariate analysis. The result was a median survival of 39 months and 5-year overall survival rate of 30%. Univariate analysis showed that surgery result (*P*=0.001), disease free interval (*P*=0.018) and the number of hepatic lesions (*P*=0.018) were significantly related to survival. Furthermore, the surgery result (*P*=0.004) remained significant for prognosis in multivariate analysis.

**Conclusions:**

For patients with OCLM, HR is safe and may provide a signiﬁcant survival beneﬁt compared with medical therapy alone. A long interval time, the number of hepatic lesions, and surgery results are key prognostic factors. Favorable outcomes can be achieved even in patients with medically controlled or surgically resectable extrahepatic disease, indicating that surgery should be considered more frequently in the multidisciplinary care of patients with OCLM.

## Introduction

Ovarian cancer, a malignant gynecologic disease, is a major cause of mortality for women^[^^1^^]^. It is the most lethal gynecological malignancy and the fifth most common cause of cancer-related deaths in women in the United States, with an estimated 21,880 new cases and 13,850 deaths in 2009^[^^2^^]^. The incidence of ovarian cancer is also steadily increasing in China^[^^3^^]^.

Although the intraperitoneal route of dissemination is considered the most common, ovarian cancer may also metastasize through the lymphatic channels and the hematogenous route^[^^4^^]^. A study by G. Cormio et al.^[^^5^^]^ reported the liver as the most common site of distant disease for ovarian cancer. Liver metastases may occur at the time of diagnosis or during the evolution of the disease. Such metastases are usually associated with widespread dissemination of the disease and poor performance status. The effects of metastases are devastating and survival is usually very poor^[^^6^^]^. This study examines our experience with hepatic resection in a relatively unselected group of patients with ovarian cancer liver metastases (OCLM).

## Patients and Methods

Eighty-seven advanced ovarian cancer patients with metachronous hepatic metastasis were admitted to the Tianjin Medical University Cancer Center Institute and Hospital from January 2001 to June 2011. All patients had undergone surgical procedures for their primary disease, usually consisting of total abdominal hysterectomy, bilateral salpingo-oophorecomy, and omentectomy ^[^^7^^]^. These patients had also received platin-based chemotherapy for the treatment of ovarian cancer. The liver metastases cases were considered resectable; all extrahepatic distant metastases (i.e., bone, lungs) were treated with stage-appropriate therapy including surgery, chemotherapy, and radiotherapy prior to hepatectomy. Clinical staging at the time of primary diagnosis was performed in all patients, based on the International Federation of Gynecology and Obstetrics (FIGO) system^[^^8^^]^. Twenty patients were turned down because their liver function or the number/distribution of metastases was not suitable for operation. Seven patients did not receive chemotherapy because of poor performance status or severe adverse reaction to chemotherapy. Sixty patients were included and thus considered for further analysis. Tumor progression was assessed every two months using CT scan, X-ray, or ultrasonic testing. PET was also available when necessary. All the data were retrospectively collected by the first author.

Univariate analysis was performed to identify predictors associated with survival rate. Cox’s proportional hazards model was performed to allow the multivariate analysis to identify factors that independently inﬂuenced survival rates. The Kaplan-Meier method was used to estimate the cumulative probabilities of the patients’ survival rate, and the differences in probabilities were evaluated using the Log-rank test. Statistical analysis was carried out using SPSS 13.0 software (SPSS Inc., Chicago, Illinois). Differences associated with *P*<0.05 were considered significant.

## Results

Sixty patients with hepatic metastasis from ovarian cancer were identified. Their median age at the time of ovarian carcinoma diagnosis was 46 years (range was from 16 to 89 years). According to the FIGO staging system, 33 patients had stage III and 27 had stage IV diseases. In the final pathological analysis of the liver specimens, most patients (*n*=54; 90.0%) showed a negative hepatic margin (R0), whereas 6 patients (10.0%) had microscopic disease at the margin (R1). Serous histology was the most common cell type, accounting for 45% of the tumors. Furthermore, the majority of patients (63.3%) showed poorly differentiated tumors. All patients received platinum/paclitaxel regimen for primary chemotherapy, with a mean cycle of 6.6±1.54. Overall, most patients (*n*=36, 60.0%) had multiple liver lesions, whereas 24 (40.0%) patients had a single metastasis in the liver. Metastases distribution was unilobar in 29 (48.3%) patients and bilobar in 31 (51.7%) patients. The median disease-free interval from initial operation to liver metastases was 34 months. Survival time was less than 12 months in 21 patients (65.0%), and more than 12 months in 39 patients (35.0%) (**Table 1**).

**Table 1:** Clinical characteristics of ovarian cancer patients with liver metastases.

Characteristics	*n*	%
Cell type		
Serous	27	45.0
Endometrioid	9	15.0
Immature teratomas	10	16.7
Granulosa cell tumor	6	10.0
Undifferentiated	8	13.3
Surgery result		
Curative (R0)	54	90.0
Palliative (R1)	6	10.0
Tumor grading		
Poor	38	63.3
Moderate	14	23.3
Well-differentiated	8	13.3
Disease-free interval		
<12 months	21	35.0
≥12 months	39	65.0
No. of liver metastases		
Single	24	40.0
Multiple	36	60.0
Extrahepatic lesions		
Yes	40	66.7
No	20	33.3
Location of liver metastases		
Unilateral	29	48.3
Bilateral	31	51.7
FIGO stage		
III	33	55.0
IV	27	45.0

Different types of hepatic resection (HR) were performed: local resection in 28 patients (46.7%), radiofrequency ablation (RFA) in 3 patients (5%), lobectomy in 7 patients (11.7%), trisegmentectomy in 7 patients (11.7%), bisegmentectomy in 12 patients (20.0%), and combined RFA in 3 patients (5%). Disease-free interval was calculated from the time of diagnosis (usually the date of primary surgery) to liver metastases, and survival time was calculated from the time of diagnosis of the liver metastases.

The sites and number of extrahepatic metastases were as follows: pleura, 10; lung and spleen, 7 each; skin, 4; extra-abdominal lymph nodes, 8; bone, 3; and breast, 1. Prior to HR, distant metastases had been diagnosed in 40 (66.7%) patients, 25 of whom were curatively resected following systemic treatment before hepatectomy. The extrahepatic metastases in the remaining 15 patients, who were well controlled with systemic therapy and local radiotherapy, underwent hepatectomy later (**Table 2**).

**Table 2:** Treatment of distant metastases by site.

Extra-hepatic lesions	*n*	Treatment
Pleura Spleen Extra-abdominal lymph nodes Bone Lungs Skin Breast	10 7 8 3 7 4 1	Surgery+chemotherapy Surgery Resection+chemotherapy+radiofrequency ablation Chemotherapy+ local radiotherapy Surgery+chemotherapy local skin excision mastectomy

The 60 patients included in the study all underwent hepatic surgery for liver metastasis. The median number of liver lesions treated was 3 (range was from 1 to 8). The majority (*n*=54; 90%) of patients underwent resection only; radiofrequency ablation only (*n*=3; 5%) and combined resection and ablation (*n*=3; 5%) were utilized much less frequently. The median postoperative days of hospital stay was seven days (range was from 3 to 24). No postoperative deaths occurred within 90 days after surgery. Six patients (10.0%) experienced postoperative complications, mostly related to wound infection or pulmonary issues. None of the patients experienced liver-related complications (i.e., biloma, abscess, liver insufficiency, or failure).

**Figure 1:**
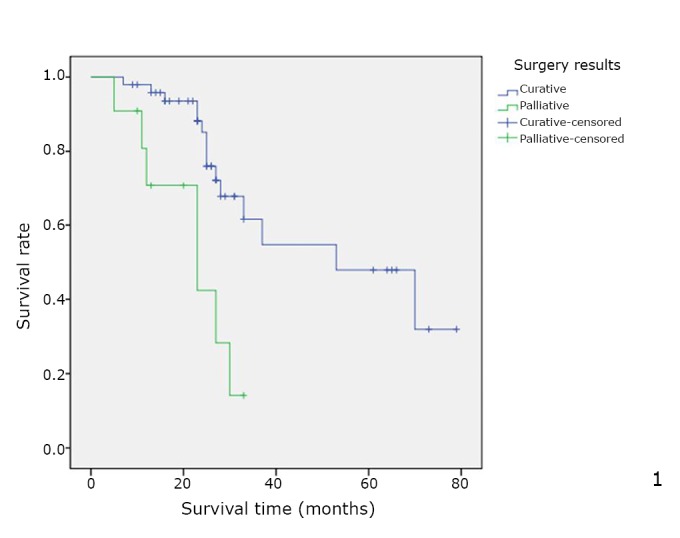
Overall cumulative survival rate for patients with OCLM undergoing resection based on curability status. Overall cumulative survival rate was significantly worse in the R1 group than in the R0 group (*P*=0.001).

**Figure 2:**
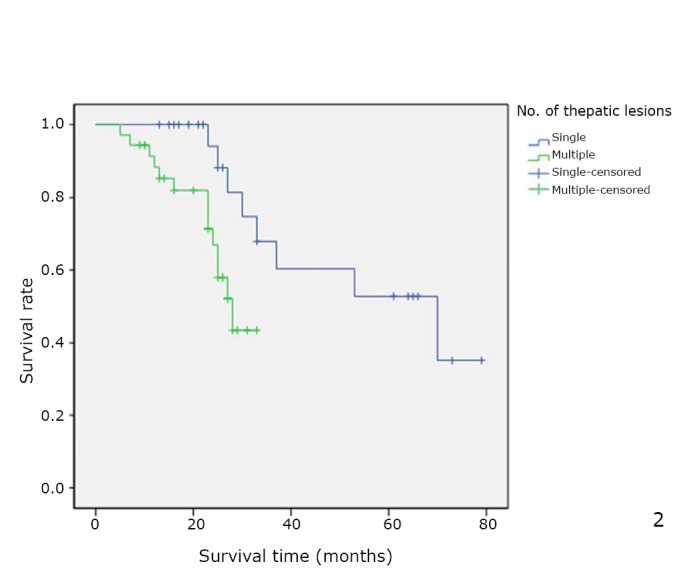
Overall cumulative survival rate for patients with OCLM undergoing resection based on the number of hepatic lesions. Overall cumulative survival rate was significantly worse in the multiple group than in the single group (*P*=0.018).

**Figure 3:**
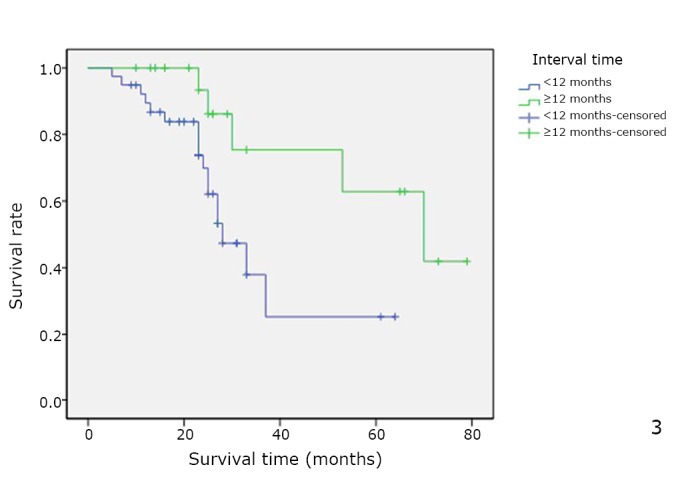
Overall cumulative survival rate for patients with OCLM undergoing resection based on disease-free interval. Overall cumulative survival rate was significantly worse in the short disease-free interval (<12 months) group than in the long disease-free interval (>12 months) group (*P*=0.018).

When calculated from the time of surgery, the median overall survival was 39 months (range was from 5 to 79), with a 5-year survival rate of 39%. In univariate analysis, surgery result (*P*=0.039, **Figure 1**), number of lesions (*P*=0.018, **Figure 2**), and disease-free interval (*P*=0.018, **Figure 3**) were the only factors significantly associated with survival. In the multivariate analysis, surgery result (*P*=0.004) also remained significant for prognosis (**Table 3**).

**Table 3:** Univariate and multivariate analysis of survival following diagnosis of distant metastases.

Characteristics	Median survival	χ^2^	Univariate *P*	Multivariate *P*
Cell type		4.45	0.349	
Serous	35.15			
Endometrioid	36.75			
Immature teratomas	30.33			
Granulosa cell tumor	47.33			
Undifferentiated	21.06			
Surgery result		10.51	0.001	0.004
Curative (R0)	51.90			
Palliative (R1)	22.01			
Tumor grading		2.00	0.368	
Poor	32.38			
Moderate	34.32			
Well-differentiated	40.81			
Disease-free interval		5.59	0.018	0.123
<12 months	30.94			
≥12 months	50.96			
No. of liver metastases		5.56	0.018	0.085
Single	55.84			
Multiple	26.06			
Extrahepatic lesions		1.12	0.290	
Yes	36.99			
No	41.42			
Location of liver metastases		0.20	0.652	
Unilateral	40.47			
Bilateral	35.45			
FIGO stage		0.33	0.563	
III	42.69			
IV	35.75			

## Discussion

Ovarian cancer remains a leading cause of death resulting from gynecologic malignancy, particularly liver metastases. However, effective chemotherapy has met with increased success at prolonging survival^[^^9^^]^. Patients with advanced disease have a response rate of more than 80% following surgery and adjuvant chemotherapy with platinum-taxane, with a median progression-free interval of 18 months^[^^10^^]^. To date, few studies have evaluated the prognostic factors associated with hepatic metastasis from ovarian cancer, resulting in scant data being reported in the literature. The current retrospective analysis evaluated 60 patients with hepatic metastasis and found that the disease-free interval, surgery result, and number of lesions were statistically significant factors associated with survival. The survival experienced by our patients, including a median survival of 39 months, compared favorably with the reported survival results for ovarian cancer patients (13 months) subjected to paclitaxel plus carboplatin treatment alone^[^^11^^]^.

The indications for surgical treatment of liver metastases are controversial, and the selection criteria for hepatectomy are not well-deﬁned. The most suitable candidates are probably those with solitary liver metastasis and no extrahepatic metastases, as the best results have been obtained in such patients. Chi et al.^[^^12^^]^ reported on a series of 12 women from Memorial Sloan-Kettering Cancer Center and concluded that patients with recurrent disease limited to certain sites in the liver can benefit from hepatic resection. Pekmezci et al.^[^^7^^]^ stated that liver resection for solitary metastases of ovarian carcinoma could be an effective and feasible approach, with a mean disease-free interval of 5.38 years from the time of initial surgery to surgery for metastatic liver lesions. Similarly, Loizzi et al.^[^^13^^]^, Kamel et al.^[^^14^^]^, and Lim et al.^[^^15^^]^ stated that selected ovarian cancers patients could benefit from liver resection of metastases.

Patients with liver metastases may have extrahepatic metastases, which is generally considered contraindicated to hepatectomy. In this study, we determined that the presence of extrahepatic disease was not a signiﬁcant prognostic factor, and that the inclusion of patients with various types of extrahepatic disease allowed the identification of a subset of patients with extrahepatic disease who experienced poor outcomes. Patients who harbored extrahepatic metastases at the time of hepatectomy had shorter overall survival time (36.99 months) than patients with no history of extrahepatic disease (41.42 months), or who either had extrahepatic disease resected or were in remission prior to hepatectomy. Although these findings were not statistically significant (*P*=0.29), both types of patients exhibited better survival than those who had undergone chemotherapy alone^[^^14^^]^. Therefore, we do not recommend that patients with stable extra-abdominal metastases be excluded from consideration for HR.

Adam et al.^[^^16^^]^ noted that favorable outcomes can be achieved even in patients with breast cancer liver metastases and medically controlled or surgically resectable extrahepatic disease, suggesting that surgery should be considered more frequently in multidisciplinary treatment. Results from the cohort study by Adam et al. showed that disease-free intervals under 12 months in duration conferred worse survival after liver metastases diagnosis than long disease-free intervals. In a previous study, Adam et al. also stated that the most important prognostic factor associated with survival is the disease-free interval between the diagnosis of ovarian cancer and the documentation of distant metastases^[^^6^^]^. By contrast, Myong et al.^[^^17^^]^ concluded that disease-free interval was not related with improved outcome. Our conclusions are in accordance with the former, which can be explained as such: the longer survival observed in patients with prolonged disease-free intervals between diagnosis of ovarian cancer and detection of liver metastasis probably reflects the biologically indolent course of such tumors.

In liver metastases from colorectal carcinoma, the number of metastases could affect the survival rate^[^^18^^]^. Patients with single metastasis have the best long-term survival, and multiple or diffuse metastases lead to the worst outcomes^[^^19^^]^. In the current study, statistically significant differences in survival were also found based on the number of ovarian cancer liver metastases in the univariate analysis.

In this study, the median overall survival from the time of liver metastasis diagnosis was 52 months for patients with negative hepatic margin (R0), compared with 22 months for patients with microscopic disease at the margin (R1), indicating that HR is an important factor significantly associated with survival. In the multivariate analysis, surgery result was the most important prognostic factor associated with survival. Standard cytoreductive procedures can increase survival rates by a statistically significant margin. Roh et al.^[^^20^^]^ and Abood et al.^[^^21^^]^ stated that hepatic resection is safe as a secondary cytoreductive surgery for recurrent ovarian cancer, and is associated with a favorable outcome in highly selected patients. This study also confirmed that hepatectomy, used as a cytoreductive treatment, might improve the survival of patients with hepatic metastases from ovarian cancer, including the stable control of extrahepatic metastases. In summary, the cancer bulk in the ovarian cancer liver metastasis is clearly a signiﬁcant prognostic factor.

None of the patients in the current study had any liver-related complications, owing to improved understanding of the hepatic anatomy and to technical advances in the last few decades^[^^22^^,^^23^^]^. Modern surgical strategies developed by major hepatobiliary centers have also demonstrated that hepatectomy of as much as 70% of the liver can be performed with a mortality rate of less than 5%^[^^24^^,^^25^^]^. Recently, a hepatobilitary surgical team at the National Cancer Center in Korea reported no operative mortalities, no major morbidities, and no reoperations related to anatomic hepatic resection in 187 patients with primary and metastatic hepatic malignancies over a 3-year period, using the new hanging maneuver^[^^26^^]^. Additionally, anatomic resections could reduce the rate of positive tumor margins and improve overall survival, according to DeMatteo et al.^[^^27^^]^.

The belief that surgical therapy has no role in the treatment of ovarian cancer patients with liver metastases is no longer valid. When included in the multimodality treatment plan, HR can be performed with low risk. HR can also improve long-term outcomes, provided that the metastatic disease responds to preoperative treatment, and that resection is radically complete. In such cases, surgical therapy can act as an effective adjuvant treatment to systemic therapies, providing selected patients with survival benefits and hope for a cure.

## References

[1] Greenlee RT Hill-Harmon MB Murray T Thun M Cancer statistics, 2001. CA Cancer J Clin 2001; 51: 15-36 11577478 10.3322/canjclin.51.1.15

[2] Jemal A Siegel R Xu J Ward E. Cancer statistics, 2010. CA Cancer J Clin 2010; 60: 277-300 20610543 10.3322/caac.20073

[3] Zhang SW Chen WQ Wei WQ Ovary cancer mortality in China 2004 - 2005: results from the Third National Retrospective Sampling Survey of Death Causes. Zhonghua Yu Fang Yi Xue Za Zhi 2010; 44: 418-422 (in Chinese) 20654231

[4] Rose PG Piver MS Tsukada Y Lau TS Metastatic patterns in histologic variants of ovarian cancer. An autopsy study. Cancer 1989; 64: 1508-1513 2776109 10.1002/1097-0142(19891001)64:7<1508::aid-cncr2820640725>3.0.co;2-v

[5] Cormio G Rossi C Cazzolla A Distant metastases in ovarian carcinoma. Int J Gynecol Cancer 2003; 13: 125-129 12657111 10.1046/j.1525-1438.2003.13054.x

[6] Dauplat J Hacker NF Nieberg RK Distant metastases in epithelial ovarian carcinoma. Cancer 1987; 60: 1561-1566 3621129 10.1002/1097-0142(19871001)60:7<1561::aid-cncr2820600725>3.0.co;2-v

[7] Pekmezci S Saribeyoglu K Aytac E Surgery for isolated liver metastasis of ovarian cancer. Asian J Surg 2010; 33: 83-88 21029944 10.1016/S1015-9584(10)60014-0

[8] Changes in definitions of clinical staging for carcinoma of the cervix and ovary: International Federation of Gynecology and Obstetrics. Am J Obstet Gynecol 1987; 156: 263-264 10681275

[9] Feldman GB Knapp RC Lymphatic drainage of the peritoneal cavity and its significance in ovarian cancer. Am J Obstet Gynecol 1974; 119: 991-994 4276313 10.1016/0002-9378(74)90021-0

[10] Yap TA Carden CP Kaye SB Beyond chemotherapy: targeted therapies in ovarian cancer. Nat Rev Cancer 2009; 9: 167-181 19238149 10.1038/nrc2583

[11] Herzog TJ Recurrent ovarian cancer: how important is it to treat to disease progression? Clin Cancer Res 2004; 10: 7439-7449 15569973 10.1158/1078-0432.CCR-04-0683

[12] Chi DS Fong Y Venkatraman ES Barakat RR Hepatic resection for metastatic gynecologic carcinomas. Gynecol Oncol 1997; 66: 45-51 9234920 10.1006/gyno.1997.4727

[13] Loizzi V Rossi C Cormio G Clinical features of hepatic metastasis in patients with ovarian cancer. Int J Gynecol Cancer 2005; 15: 26-31 15670293 10.1111/j.1048-891x.2005.14406.x

[14] Kamel SI de Jong MC Schulick RD The role of liver-directed surgery in patients with hepatic metastasis from a gynecologic primary carcinoma. World J Surg 2011; 35: 1345-1354 21452068 10.1007/s00268-011-1074-y PMC3568526

[15] Lim MC Park SY Seo SS E-NOTES: Promising minimal surgical approach for gynecologic disease. Gynecol Oncol 2009; 115: 320-321; author reply 321 19698978 10.1016/j.ygyno.2009.07.018

[16] Adam R, Aloia T, Krissat J, et al. Is liver resection justified for patients with hepatic metastases from breast cancer? Ann Surg 2006; 244: 897-907; discussion 907-898. 10.1097/01.sla.0000246847.02058.1b PMC1856635 17122615

[17] Lim MC Kang S Lee KS The clinical significance of hepatic parenchymal metastasis in patients with primary epithelial ovarian cancer. Gynecol Oncol 2009; 112: 28-34 19010521 10.1016/j.ygyno.2008.09.046

[18] Fujita S Akasu T Moriya Y. Resection of synchronous liver metastases from colorectal cancer. Jpn J Clin Oncol 2000; 30: 7-11 10770561 10.1093/jjco/hyd002

[19] Wyld L Gutteridge E Pinder SE Prognostic factors for patients with hepatic metastases from breast cancer. Br J Cancer 2003; 89: 284-290 12865918 10.1038/sj.bjc.6601038 PMC2394248

[20] Roh HJ Kim DY Joo WD Hepatic resection as part of secondary cytoreductive surgery for recurrent ovarian cancer involving the liver. Arch Gynecol Obstet 2011; 284: 1223-1229 21132314 10.1007/s00404-010-1750-4

[21] Abood G Bowen M Potkul R Hepatic resection for recurrent metastatic ovarian cancer. Am J Surg 2008; 195: 370-373 18207130 10.1016/j.amjsurg.2007.12.012

[22] Shah SA Bromberg R Coates A Survival after liver resection for metastatic colorectal carcinoma in a large population. J Am Coll Surg 2007; 205: 676-683 17964443 10.1016/j.jamcollsurg.2007.06.283

[23] Gold JS Are C Kornprat P Increased use of parenchymal-sparing surgery for bilateral liver metastases from colorectal cancer is associated with improved mortality without change in oncologic outcome: trends in treatment over time in 440 patients. Ann Surg 2008; 247: 109-117 18156930 10.1097/SLA.0b013e3181557e47

[24] Jarnagin WR Gonen M Fong Y Improvement in perioperative outcome after hepatic resection: analysis of 1,803 consecutive cases over the past decade. Ann Surg 2002; 236: 397-406 12368667 10.1097/01.SLA.0000029003.66466.B3 PMC1422593

[25] Chang YC Low mortality major hepatectomy. Hepatogastroenterology 2004; 51: 1766-1770 15532822

[26] Kim SH Park SJ Lee SA Various liver resections using hanging maneuver by three glisson’s pedicles and three hepatic veins. Ann Surg 2007; 245: 201-205 17245172 10.1097/01.sla.0000245516.10349.c5 PMC1876991

[27] DeMatteo RP Palese C Jarnagin WR Anatomic segmental hepatic resection is superior to wedge resection as an oncologic operation for colorectal liver metastases. J Gastrointest Surg 2000; 4: 178-184 10675241 10.1016/s1091-255x(00)80054-2

